# Characteristics of Fungal Communities in Lava Plateau Ecosystems

**DOI:** 10.3390/microorganisms14030642

**Published:** 2026-03-12

**Authors:** Yanli Zhang, Yan Zhu, Jiaxing Huang, Jiaxin Xue, Yiwei Liu, Haocong Li, Lingjie Shi, Jianhui Jia, Yueyu Sui

**Affiliations:** 1 School of Life Science and Technology, Mudanjiang Normal University, Mudanjiang 157011, China; 2 State Key Laboratory of Black Soils Conservation and Utilization, Northeast Institute of Geography and Agroecology, Chinese Academy of Sciences, Harbin 150081, China

**Keywords:** lava plateau, vegetation type, fungi, community structure, co-occurrence network, LEfSe analysis, functional prediction

## Abstract

Soil fungi are pivotal drivers of biogeochemical cycling, mediating nutrient transformation, plant–soil feedbacks, and ecosystem stability. Understanding their responses to vegetation succession is essential for predicting ecosystem recovery in fragile volcanic landscapes. We investigated soil fungal communities across five successional stages on the Jingpo Lake lava plateau—grassland (GL), shrubland (SL), deciduous broad-leaved forest (DB), coniferous and broad-leaved mixed forest (CB), and coniferous forest (CF)—using high-throughput ITS sequencing and soil physicochemical analysis. Basidiomycota and Ascomycota dominated at the phylum level, with *Sebacina*, *Cortinarius*, and *Mortierella* as core genera. Alpha diversity (Shannon, Simpson, Chao1) was significantly higher in early-successional GL and SL than in DB (*p* < 0.05), while CB exhibited the lowest community evenness (Pielou-e). Co-occurrence networks revealed greater connectivity in GL, whereas forest types showed simplified topologies. LEfSe identified distinct fungal biomarkers for each vegetation type. PICRUSt2-based functional prediction indicated biosynthesis as the dominant pathway (>40%), with significant variation among vegetation types. Redundancy analysis (RDA) identified soil organic matter (SOM) as the primary predictor of fungal community composition. Our findings indicate that vegetation succession is associated with changes in fungal diversity and function primarily linked to variations in SOM, with moisture regimes as a secondary contextual factor. Notably, advanced forest stages exhibited reduced fungal diversity and simplified community structure—highlighting a trade-off between nutrient enrichment and microbial complexity in volcanic ecosystems. These insights advance our understanding of plant–soil–microbe coupling during ecosystem restoration on lava plateaus.

## 1. Introduction

Soil microorganisms drive biogeochemical cycles and thus mediate soil–atmosphere exchange, enhance soil fertility, and sustain ecosystem function stability [1]. Fungi, major biological components of soil ecosystems, are particularly important for plant growth, development, and environmental adaptation. Mycorrhizal fungi, the most active group within the soil fungal community, increase plant productivity and provide resistance to biotic and abiotic stresses [2]. Additionally, as the dominant soil microbial group, saprotrophic fungi degrade and transform complex organic matter, thereby serving as key indicators for assessing ecological conditions [3]. Therefore, the structure and diversity of soil fungal communities are decisive factors for soil quality and vegetation health. Vegetation types and soil environmental factors—such as pH, moisture content, organic matter, and total nitrogen—are the primary drivers that shape the composition and diversity of the soil fungal community across grasslands, forests, wetlands, and other ecosystems [4,5,6,7].

Volcanic lava plateaus are distinctive natural landscapes that are primarily composed of basalt [8]. Their formation and subsequent evolution influence vegetation, soil, and microbial communities. Soil development on these plateaus is governed by environmental factors such as climate, precipitation, and weathering, as well as by the structural characteristics of the bedrock [9]. As rocks weather, differences in mineral composition produce spatially distinct soil types. In particular, juvenile volcanic lava soils and dark-brown juvenile soils on lava plateaus show a spatial heterogeneity that underlies the differentiation of soil genetic horizons. This heterogeneity also drives vegetation succession and niche partitioning by creating diverse habitat substrates [10].

Jingpo Lake is a landmark ecological area in Heilongjiang Province surrounded by a unique and sensitive volcanic lava plateau ecosystem. As a key area for studying Cenozoic inland volcanoes, the Jingpo Lake lava plateau is a natural base for other studies of numerous scientific issues in volcanic ecosystems such as the flora of communities, soil nutrients, and soil microbial communities [11]. Its unique geographical morphology and rock characteristics lay the foundation for soil development and vegetation succession [12]. Currently, most studies on the characteristics of soil microbial communities focus on typical ecosystems such as loess hills, alpine meadows, and karst landscapes, while there are relatively few reports on volcanic lava plateau areas with fragile ecological environments and exposed rocks [13]. To address this gap, this study aims to comprehensively characterize the multidimensional features of soil fungal communities—including taxonomic composition, α-diversity patterns, co-occurrence network topology, functional potential, and key environmental drivers—across a vegetation succession sequence on the Jingpo Lake lava plateau. Therefore, in this study, five vegetation types, namely grassland, shrubland, deciduous broad-leaved forest, coniferous and broad-leaved mixed forest, and coniferous forest, on the lava plateau of Jingpo Lake were selected as research objects according to their succession processes. The soil fungal community structure was analyzed to identify the dominant fungal communities. By considering the associations between the physical and chemical properties of the soil and the characteristics of the soil fungal community together, the diversity of these communities in different vegetation types and the driving factors behind this diversity were revealed. The roles of soil fungi in material cycling and energy flow were clarified, providing a theoretical reference for vegetation restoration and sustainable development of the lava plateau ecosystem.

## 2. Materials and Methods

### 2.1. Site Profile

**Figure 1:**
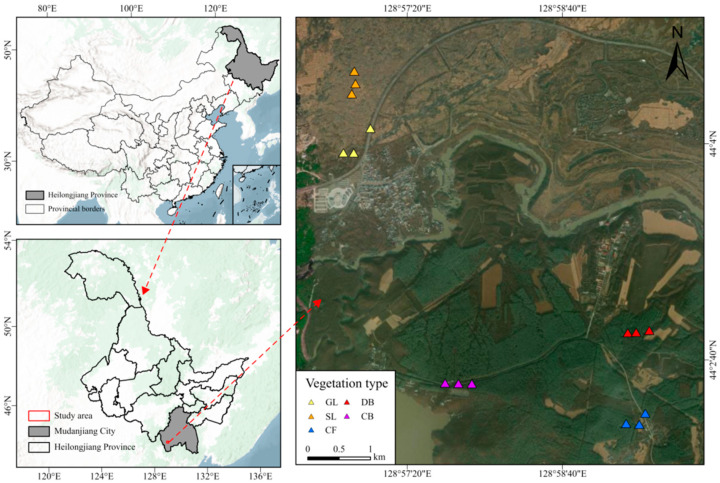
Overview of Jingpo Lake National Geopark.

The Jingpo lava plateau, northeast China, is located at 128°30′–129°10′ E and 44°0′–44°20′ N (Figure 1). A special type of landform, lava plateaus are closely related to volcanic activity. Specifically, after the lava cools and solidifies, due to terrain uplift and erosion, a “table-like” landform with a flat surface and steep edges is formed. The total area of the basalt lava plateau is 200 km^2^, with a forest coverage rate of over 68%. The volcanic eruptions occurred between 5200 and 5500 years ago. The study area has a mid-temperate continental monsoon climate, featuring distinct seasonal climate characteristics. The average annual temperature is only 4.3 °C, and the average annual precipitation is 619.8 mm. The main soil types are alkaline volcanic lithosol and dark-brown volcanic ash soil.

### 2.2. Soil Collection and Treatment

Sampling was performed in July 2023 across five vegetation types: grassland (GL), shrubland (SL), deciduous broad-leaved forest (DB), coniferous and broad-leaved mixed forest (CB), and coniferous forest (CF). Figure 1 details the sampling site locations. A 10 m × 10 m quadrat was established, with 1 m × 1 m quadrats placed at each corner and the center for community surveys. Dominant species are listed in Table 1. Three replicates were set up for each vegetation type, resulting in 15 samples total. Subsequent experiments such as analyses of soil physicochemical properties and microbial sequencing were carried out using the replicated design. Soil was collected from the 0–10 cm depth using sterile stainless steel soil augers, ensuring a slope change of less than 5°. At each sampling point, approximately 50 g of soil was collected with a sterile trowel, resulting in a total of 250 g per replicate after mixing five points arranged in an X-pattern. Immediately after collection, the soil was placed in sterile polypropylene bags and transported to the laboratory on ice. One portion (100 g) was air-dried at room temperature for physicochemical analysis, while the remaining portion (150 g) was immediately frozen at −20 °C in the field and subsequently stored at −80 °C for microbial DNA extraction and sequencing.

### 2.3. Determination of the Physical and Chemical Properties of Soil

Soil bulk density (BD) was determined employing the ring-knife weighing technique [14], while soil moisture content (SMC) was assessed through oven drying [15]. Soil pH was measured using electrode potential [16], and soil organic matter (SOM) was quantified using potassium dichromate with external heating. Total nitrogen (TN) was analyzed using the Kjeldahl method [17] and total phosphorus (TP) was determined through digestion, while total potassium (TK) was evaluated utilizing the NaOH fusion flame photometry method [18]. Available nitrogen (AN) was assessed via alkaline hydrolysis diffusion, available phosphorus (AP) was extracted with a 0.5 mol/L NaHCO_3_ solution [19], and available potassium (AK) was measured using an extraction method using 1 mol/L NH_4_OAc and 0.01 mol/L EDTA [20,21,22].

### 2.4. DNA Extraction, PCR Amplification, and Amplicon Sequencing

Soil microbial DNA was extracted using the MoBio PowerSoil^®^ DNA Isolation Kit (Product No. 12888-100, MO BIO Laboratories, Carlsbad, CA, USA). DNA quality was assessed via 1% agarose gel electrophoresis, and quantity was determined using a NanoDrop One UV-Vis Spectrophotometer (Thermo Fisher Scientific, Waltham, MA, USA). The fungal ITS1 region was amplified with primers F (5′-GAACCWGCGGARGGATCA-3′) and R (5′-GCTGCGTTCTTCATCGATGC-3′). PCR products were visualized on 2% agarose gel and purified with AMPure XT beads (Beckman Coulter Genomics, Danvers, MA, USA). Subsequently, purification was performed using AMPure XP beads (Beckman Coulter Genomics, Danvers, MA, USA) to remove primers and nonspecific fragments. Amplicons were quantified using an Agilent 210 Bioanalyzer (Agilent, Santa Clara, CA, USA) and Illumina library quantification kits (Kapa Biosciences, Wilmington, MA, USA). Sequencing was performed on the Illumina NovaSeq 6000 (PE250) platform manufactured by Hangzhou LC-Bio Technology Co., Ltd. (Hangzhou, China), generating paired-end reads of 2 × 250 bp, with an average of 80,000 reads per sample.

Quality filtering was performed to ensure sufficient sequencing depth. Specifically, sequences were retained only if they met the following criteria: (i) Phred quality score ≥ 30 (Q30, corresponding to a base-calling error rate ≤ 0.1%); (ii) merged read length between 200 and 400 bp; (iii) no ambiguous bases (N); and (iv) no chimeric sequences as identified by DADA2’s built-in chimera removal algorithm. Negative controls were integrated into both DNA extraction and sequencing processes to monitor potential contamination.

The fungal sequencing data underwent bioinformatics analysis with QIIME 2 (v 2023.09) [23]. DADA2 was utilized via ‘qiime dada2 denoise-paired’ for quality trimming, noise reduction, sequence merging, chimera detection, and ASV (Amplicon Sequence Variant) clustering. Species annotation was performed using the RDP Classifier (https://sourceforge.net/projects/rdp-classifier/, accessed on 1 January 2026) [23] and unite (https://unite.ut.ee/, accessed on 3 January 2026) [24] databases, with statistical analysis of species abundance per sample based on the ASV abundance table. The annotation confidence threshold was established at 0.7.

Endophytic fungi were functionally predicted and visualized based on PICRUSt2 (https://github.com/picrust/picrust2, accessed on 3 January 2026) using the Microeco bioinformatics cloud (https://www.bioincloud.tech/, accessed on 4 January 2026) and STAMP software (statistical analysis of taxonomic and functional profiles, V2.1.3). This approach enables high-resolution functional profiling when metagenomic sequencing is unavailable.

### 2.5. Data Processing

Statistical analyses were performed using IBM SPSS Statistics 27.0 and R (v4.3.1), with data visualization conducted in Origin 2021 (https://www.originlab.com/, accessed on 1 February 2026). Normality and homoscedasticity were assessed using Shapiro–Wilk and Levene’s tests, respectively; variables violating these assumptions were log(x + 1)-transformed prior to parametric testing.

Prior to diversity and network analyses, the ASV table was rarefied to the minimum sequencing depth of 82,551 reads per sample to ensure comparability among samples. This rarefaction threshold was determined based on the sample with the lowest valid read count after quality filtering, ensuring that all samples retained sufficient sequencing depth for robust diversity estimation (Coverage index > 97% for all samples).

One-way ANOVA with Tukey’s HSD post hoc test (α = 0.05) was used to compare (i) soil physicochemical properties (BD, SMC, pH, SOM, TN, TP, TK, AN, AP, AK), (ii) fungal alpha diversity indices (Shannon, Simpson, Chao1, Pielou-e, Coverage), and (iii) relative abundances of dominant fungal taxa (>1%) across vegetation types. Categorical variable distributions were evaluated using Chi-square tests.

LEfSe analysis was performed via the Majorbio Cloud Platform (https://www.majorbio.com/, accessed on 4 February 2026) with Kruskal–Wallis α = 0.05 and LDA score threshold = 2.0.

Co-occurrence networks were constructed using Spearman correlations (|r| > 0.6, *p* < 0.05, FDR-corrected) via the Majorbio Cloud Platform and visualized in Gephi 0.10.1 (https://gephi.org/, accessed on 5 February 2026); topological differences among vegetation types were descriptively compared (*n* = 3 per group).

Pearson correlation coefficients with FDR correction for multiple testing (significance threshold: *p* < 0.05) were calculated between soil physicochemical properties and fungal community composition/diversity via the Lianchuan BioCloud platform (https://www.omicstudio.cn/, accessed on 7 February 2026). Redundancy analysis (RDA) was conducted using CANOCO 5 (https://www.Canoco5.com, accessed on 7 February 2026) to visualize the relationships between fungal community composition and environmental variables. Prior to model construction, detrended correspondence analysis (DCA) confirmed a linear response model (gradient length < 3.0 SD). Environmental variables were standardized, and multicollinearity was assessed using Variance Inflation Factors (VIFs); variables with VIF > 10 were excluded to ensure model robustness. Significant predictors were identified via forward selection to determine independent explanatory variables with 999 permutation tests (FDR-corrected, *p* < 0.05).

Functional potential of fungal communities was predicted using PICRUSt2 based on the MetaCyc database; results represent inferred metabolic capacity rather than in situ enzymatic activity.

Significance threshold for all tests: *p* < 0.05.

## 3. Results

### 3.1. Physicochemical Properties of Soils with Different Vegetation Types

Soil physical and chemical properties were determined following the methods described in our previous study [11]. The results differed significantly among vegetation types (One-way ANOVA, *p* < 0.05; Table 2). BD was highest in GL and lowest in CF, with the BD of SL and GL significantly greater than those in forest vegetation types (CF, CB, and DB) (*p* < 0.05). The SMC displayed the opposite trend to BD, with SMC values in forest vegetation types significantly higher than those in SL and GL (*p* < 0.05). Soil pH was weakly acidic for all vegetation types on the Jingpo lava plateau. GL, which features sparse vegetation cover, differs significantly from CF (*p* < 0.05), while other vegetation types show no obvious differences. SOM, TN, TP, and TK varied by vegetation type in the order CF > CB > DB > SL > GL. AN, AP, and AK display similar patterns across vegetation types.

### 3.2. Composition of Soil Fungal Communities Under Different Vegetation Types

**Figure 2:**
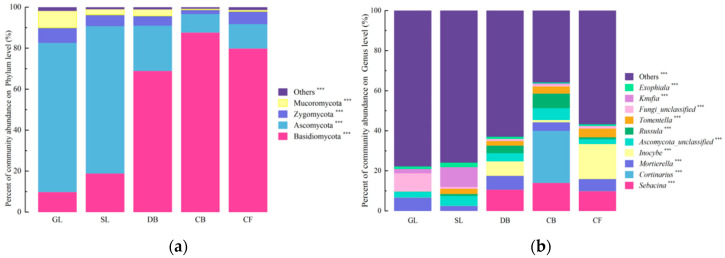
Community composition of soil fungal communities across five vegetation types on the Jingpo Lake lava plateau. (**a**) Relative abundance of fungal phyla with abundance > 1% (four dominant phyla); the “Others” category includes the remaining 10 phyla with relative abundance ≤ 1%. (**b**) Relative abundance of the top 10 most abundant genera (out of 23 genera with relative abundance > 1%); the “Others” category includes the remaining 13 genera with relative abundance > 1% plus all genera with relative abundance ≤ 1%. Asterisks denote significant differences in relative abundance among vegetation types based on one-way ANOVA with Tukey’s HSD post hoc test: *** *p* < 0.001. Error bars represent standard deviation (SD); *n* = 3 per vegetation type.

High-throughput sequencing of the fungal ITS gene yielded 1,180,590 valid sequences, which were taxonomically annotated against the RDP database. At the phylum level (Figure 2a), 14 phyla were identified. Among these, four phyla with a relative abundance > 1%—Basidiomycota, Ascomycota, Zygomycota, and Mucoromycota—accounted for 98.77% of the total fungal community. The relative abundances of these four dominant phyla differed significantly across the five vegetation types (*p* < 0.05). At the genus level (Figure 2), 23 genera with a relative abundance > 1% were detected. Figure 2b displays the top 10 most abundant genera (out of 23 genera with relative abundance > 1%). The dominant genera were *Sebacina*, *Cortinarius*, and *Mortierella*. The “Others” category includes the remaining 13 genera (>1% abundance) and all genera ≤ 1% abundance. Specifically, the relative abundances of *Sebacina*, *Cortinarius*, *Ascomycota_unclassified*, and *Russula* were significantly higher for CB vegetation than under the other four types (*p* < 0.05), while *Inocybe* exhibited a significantly higher relative abundance under DB vegetation (*p* < 0.05).

### 3.3. Diversity of Soil Fungal Communities Under Different Vegetation Types

Alpha diversity indices were compared among vegetation types using One-way ANOVA with Tukey’s HSD post hoc test (α = 0.05). Table 3 demonstrates that the Shannon and Simpson indices in GL and SL were significantly higher than in DB and CB (Tukey’s HSD, *p* < 0.05). Similarly, the Chao1 index in GL and SL surpassed that in DB (*p* < 0.05). Soil fungi in GL exhibited the greatest species number, community abundance, and diversity. The coverage indices for all five vegetation types exceeded 97%, confirming that the sequencing results accurately reflected the fungal community composition. The sequencing depth was adequate to encompass most microorganisms, including rare species; thus, the true fungal scenario is represented. The Pielou-e index for CB was significantly lower than that for SL (*p* < 0.05), indicating marked unevenness in the fungal community distribution in CB, characterized by low species diversity and a simplified community structure.

**Table 3:** Characterization of soil fungal richness and diversity indices under different vegetation types.

	Shannon Index	Simpson Index	Chao1 Index	Pielou-e Index	Coverage Index
GL	7.41 ± 0.22 ^a^	0.98 ± 0.01 ^a^	843.73 ± 45.04 ^a^	0.78 ± 0.01 ^ab^	0.97 ± 0.01 ^a^
SL	7.17 ± 0.11 ^a^	0.97 ± 0.01 ^a^	799.20 ± 3.37 ^a^	0.79 ± 0.01 ^a^	0.98 ± 0.02 ^a^
DB	4.97 ± 0.14 ^c^	0.92 ± 0.01 ^b^	369.34 ± 9.96 ^d^	0.68 ± 0.01 ^c^	0.97 ± 0.03 ^a^
CB	4.84 ± 0.21 ^c^	0.92 ± 0.02 ^b^	431.76 ± 29.22 ^c^	0.65 ± 0.02 ^d^	0.97 ± 0.01 ^a^
CF	6.15 ± 0.08 ^b^	0.94 ± 0.05 ^ab^	644.06 ± 37.59 ^b^	0.76 ± 0.03 ^b^	0.98 ± 0.01 ^a^

Note: The first column in the table shows the type of sample site. GL (grassland), SL (shrubland), DB (deciduous broad-leaved forest), CB (coniferous and broad-leaved mixed forest), CF (coniferous forest).

### 3.4. Co-Occurrence Network Patterns of Soil Fungal Communities in Different Vegetation Types

**Figure 3:**
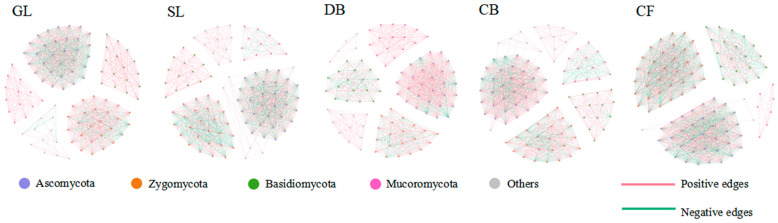
Co-occurrence network of soil fungal communities in different vegetation types. Networks were constructed based on Spearman correlations (|r| > 0.6, *p* < 0.05, FDR-corrected) among the top 100 most abundant ASVs. Node size represents relative abundance; edge color indicates positive (red) or negative (green) correlations. Sample size: *n* = 3 per vegetation type.

For the soil fungal community, the core microbiome was defined as the top 100 fungal ASVs based on their abundance across all samples. A complete fungal network was constructed by conducting co-occurrence network analysis on these 100 dominant fungal ASVs (Figure 3). The results show that the network topologies of soil fungal communities under different vegetation types exhibited distinct patterns. The network topology structure (Table 4) shows that the number of nodes in the five sample plots is basically the same, but the total number of connections, average degree, and average clustering coefficient were numerically highest for GL. The average path length of DB is the longest. The positive correlation ratios in the co-occurrence networks of the five vegetation types are all greater than the negative correlation ratios. In contrast, the modular values of the empirical network (i.e., the fungal co-occurrence network constructed in this study) are relatively high, ranging from 0.624 to 0.713, indicating that the network has significant modular structural characteristics.

### 3.5. Differential Analysis of Soil Fungal Communities in Different Vegetation Types

**Figure 4:**
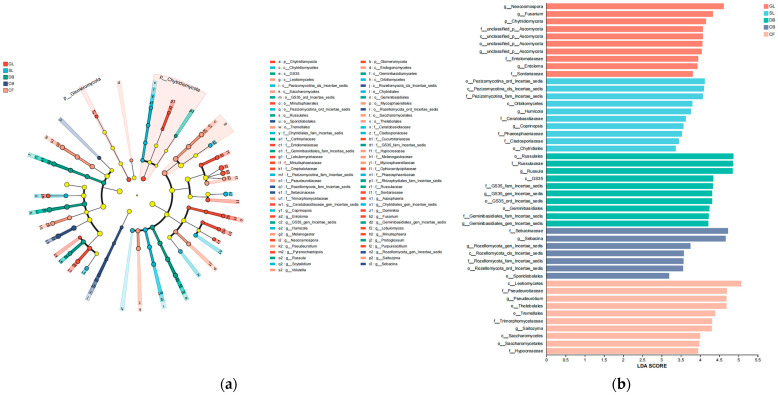
(**a**) Phylogenetic diagrams of soil fungi in different vegetation types. (**b**) Bar chart of LDA value distribution of soil fungi in different vegetation types. Differential taxa were identified using LEfSe analysis with Kruskal–Wallis test (α = 0.05) and LDA score threshold = 2.0. Sample size: *n* = 3 per vegetation type.

Five types of fungal communities were identified via the linear discriminant analysis effect size (LEfSe) method, and the core fungal communities with statistical significance are shown in Figure 4a. The figure shows five taxonomic groups with significant differences between phylum and genus. The LEfSe results show that the fungal community contains 47 branches (1 phylum, 7 classes, 11 orders, 15 families, and 13 genera). Furthermore, the results of the linear discriminant analysis (LDA) (Figure 4b) indicate that the numbers of fungal groups within the five vegetation types were 10, 10, 10, 7, and 10. This suggests that there are differences in the fungal composition in soils of different vegetation types, with CB containing the fewest biomarkers.

### 3.6. Prediction of Fungal Community Function in Different Vegetation Types

**Figure 5:**
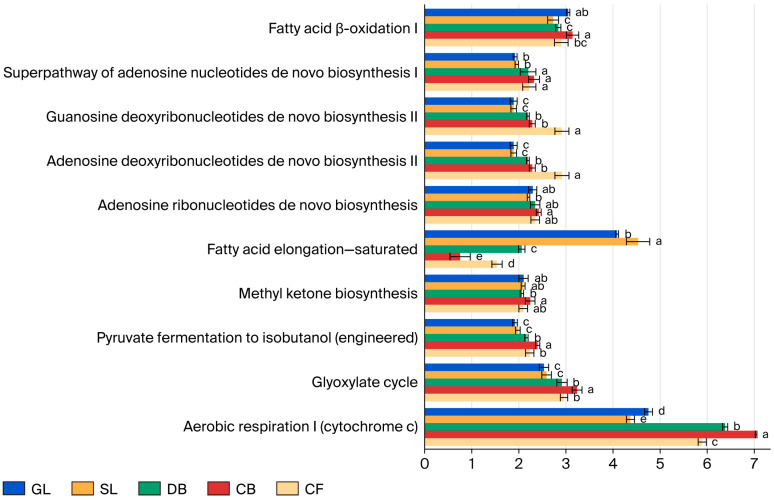
Functional predictions of soil fungal communities under different vegetation types based on the MetaCyc database using PICRUSt2 (only the top 10 pathways with relative abundance > 1% are shown). Different lowercase letters (a, b, c, d, e) above bars indicate significant differences (*p* < 0.05) in relative abundance of each metabolic pathway among vegetation types based on one-way ANOVA with Tukey’s HSD post hoc test. Error bars represent standard deviation (SD); *n* = 3 per vegetation type.

Functional potential of soil fungal communities across various vegetation types was predicted using PICRUSt2 based on the MetaCyc database (Figure 5). It should be noted that these results represent inferred metabolic capabilities based on marker gene data rather than direct measurements of in situ enzymatic activity. The analysis identified five primary metabolic pathways and 87 secondary metabolic functions across the five vegetation types. The primary pathways include biosynthesis, degradation/utilization/assimilation, the generation of precursor metabolites and energy, superpathways, and metabolizers. Notably, biosynthesis constitutes over 40% of the primary pathways, serving as the predominant predicted function of the soil fungal community in the study area. Among the secondary functions, 48 exhibited a relative abundance exceeding 1%. These included 20 principal sub-functions linked to biosynthesis, 10 linked to degradation/utilization/assimilation, 10 linked to superpathways, and 8 linked to the generation of precursor metabolites and energy. Significant variations were observed among the five vegetation types (*p* < 0.05) in aerobic respiration I (Cytochrome c) within the generation of precursor metabolites and energy and saturated fatty acid elongation in biosynthesis. The pathways for generating precursor metabolites and energy were significantly more pronounced under CB compared to other vegetation types (*p* < 0.05), while superpathways for GL and SL were notably lower than those in forest vegetation types (*p* < 0.05).

### 3.7. Analysis of the Correlation Between Soil Physical and Chemical Properties and Fungal Community Composition and Diversity of Different Vegetation Types

**Figure 6:**
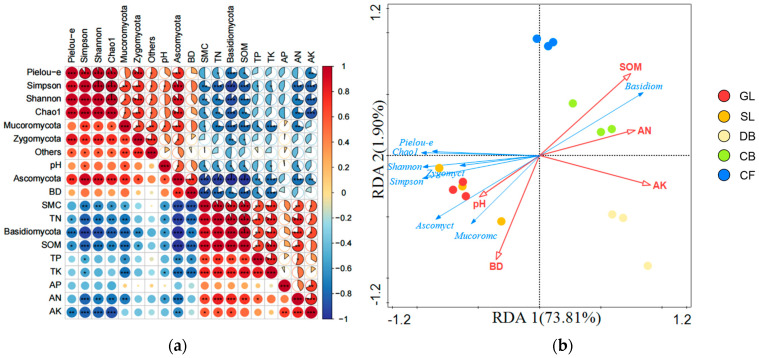
(**a**) Correlation between soil physicochemical properties and the composition and diversity of fungal phylum communities. Correlations were calculated using Pearson correlation coefficients with FDR correction for multiple testing: *** *p* < 0.001; ** *p* < 0.01; * *p* < 0.05; *n* = 15 samples. (**b**) Redundancy analysis (RDA) of soil fungal communities, diversity, and environmental variables at the phylum level *(n* = 15 samples). The model was validated by forward selection with 999 permutation tests (FDR-corrected *p* < 0.05). Red arrows indicate significant environmental predictors; blue arrows represent fungal phyla and diversity indices; circles represent sample locations.

The physical and chemical properties of soil are significantly correlated with the composition and diversity of fungal communities (Figure 6). In terms of community composition (Figure 6a), SMC, SOM, TN, TK, and AN were all extremely significantly positively correlated with Basidiomycota (*p* < 0.01), and TP and AK were significantly positively correlated with Basidiomycota (*p* < 0.05). Ascomycota were extremely significantly positively correlated with BD (*p* < 0.01), significantly positively correlated with pH (*p* < 0.05), and extremely significantly negatively correlated with SMC, SOM, TN, TK, and AN (*p* < 0.01). In terms of diversity indices (Figure 6a), the Shannon index was extremely significantly negatively correlated with SOM and AK (*p* < 0.01) and significantly negatively correlated with SMC, TN, and AN (*p* < 0.05). The Simpson index was significantly positively correlated with pH (*p* < 0.05), extremely significantly negatively correlated with SOM, TN, AN, and AK (*p* < 0.01), and significantly negatively correlated with SMC (*p* < 0.05). The Chao1 index was extremely significantly negatively correlated with AK (*p* < 0.01) and significantly negatively correlated with SMC, SOM, TN, and AN (*p* < 0.05). Since DCA1 = 1.2 < 3.0, RDA was performed (forward selection, 999 permutation tests, FDR-corrected *p* < 0.05) to analyze the relationship among soil physicochemical properties, vegetation types, and fungal communities, with fungal phyla and diversity as response variables and soil physicochemical properties as explanatory variables (Figure 6b). RDA1 explained 73.81% of the variation, while RDA2 explained 1.90% of the variation. Among the properties, SOM was identified through forward selection as the primary predictor, exhibiting the greatest explanatory power for the structure and diversity of soil fungal communities (R^2^ > 0.90, *p* < 0.01), suggesting a strong association with community variation relative to other measured soil properties.

### 3.8. Correlation Between Soil Physical and Chemical Properties and Fungal Community Function in Different Vegetation Types

**Figure 7:**
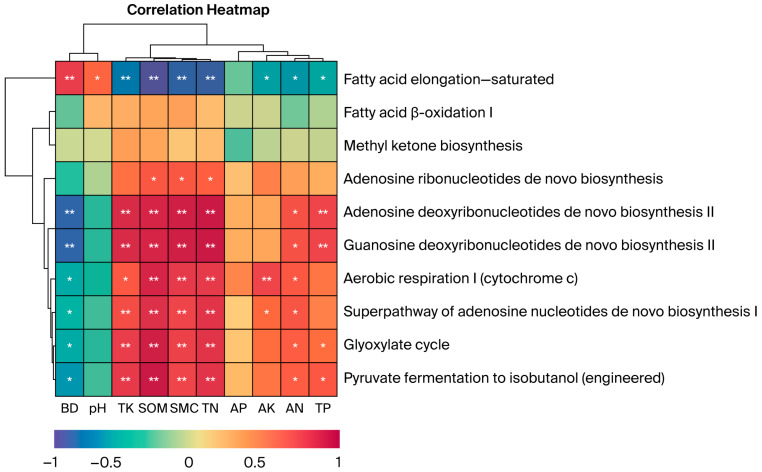
Correlations between soil physicochemical factors under different vegetation types and functional relative abundance (only the top 10 functions with relative abundance > 1% are shown). Correlations were calculated using Pearson correlation coefficients with FDR correction for multiple testing: ** *p* < 0.01; * *p* < 0.05. Sample size: *n* = 15 samples (3 replicates × 5 vegetation types). Error bars represent standard deviation (SD).

Pearson correlation analysis with hierarchical clustering was performed to examine relationships between soil physicochemical properties and functional relative abundance. The metabolic functions of soil fungal communities exhibited varied correlations with the soil’s physical and chemical properties (Figure 7). Fatty acid elongation—saturated showed highly significant correlations with BD, TK, SOM, SMC, and TN (*p* < 0.01) and significant correlations with pH, AK, AN, and TP (*p* < 0.05). Both adenosine and guanosine deoxyribonucleotide de novo biosynthesis II were highly correlated with BD, TK, SOM, SMC, TN, and TP (*p* < 0.01) and significantly correlated with AN (*p* < 0.05). Aerobic respiration I (Cytochrome c) was significantly correlated with BD, TK, and AN (*p* < 0.05) and highly significantly correlated with SOM, SMC, TN, and AK (*p* < 0.01). Fermentation of pyruvate to isobutanol (engineered) and the glyoxylate cycle exhibited significant correlations with AN, TP, and BD (*p* < 0.05) and highly significant correlations with TK, SOM, SMC, and TN (*p* < 0.01). The superpathway of adenosine nucleotide de novo biosynthesis I was highly significantly correlated with BD, AK, and AN (*p* < 0.01). The dendrogram illustrates the clustering relationships among biochemical processes, where similar processes are grouped and distinct metabolic pathways and soil physicochemical properties are clustered. These diagrams are constructed using the strength and pattern of the correlations. The results confirm the established link between C and N cycling and resource availability, showing that metabolic pathways often change concurrently under similar soil conditions. This suggests they may be driven by similar environmental factors or exhibit synergistic effects in certain ecological processes.

## 4. Discussion

### 4.1. Soil Physicochemical Properties Across Vegetation Types

The physical and chemical properties of soil are core parameters for evaluating soil quality and fertility [25]. Differences in soil physicochemical properties among vegetation types in lava plateau ecosystems mainly stem from multiple factors, such as soil parent material, weathering degree, leaching intensity, and human activities [26]. This study showed that the SOM content of forest vegetation was significantly higher than that of SL and GL. The trend in soil bulk BD was opposite to that of SOM content, consistent with the findings of Li Qinglin [27], Pang Shengjiang [28], etc., on karst landforms. This might be because forest vegetation root systems are relatively well developed, making soil structure loose and porous, which is conducive to SOM accumulation and reduced BD. In addition, the trend in SMC paralleled that of SOM content, similar to previous research [29]. Due to different root characteristics and soil utilization modes, GL vegetation exhibited relatively high BD and low SMC. In contrast, forest vegetation has lush branches and leaves that block solar radiation and rainfall, reducing soil water evaporation, enhancing water retention capacity, and weakening splash erosion. Forest vegetation is associated with enhanced SMC alongside improvements in soil structure and increased SOM. Soil pH plays a crucial role in regulating nutrient cycling [30]. Notably, pH in GL and SL was significantly higher than in forests, likely due to litter accumulation and microbial decomposition, which reduce pH through organic matter mineralization and organic acid release. CF exhibited the lowest pH, since its litter contains substances such as tannins and resins that increase acidity during decomposition. In addition, CF litter, which has a small specific leaf area and thick cuticle, decomposes more slowly than DB litter [31]. Total and available soil nutrients varied markedly among vegetation types. In forests, surface soil nutrient concentrations increase through litter decomposition and root turnover. Plant growth, root and microbial metabolism, and debris decomposition convert insoluble compounds into forms accessible to plants, thereby increasing plant-available nutrients. These findings align with previous research [28]. Overall, soil physicochemical properties interact across vegetation types and jointly enhance soil quality through complex biogeochemical cycles.

### 4.2. Fungal Community Composition and Alpha Diversity

Soil fungal communities were similar across vegetation types at the phylum level but differed in relative abundance. Volcanic eruptions have been reported to alter soil microbial community structure [32], and dominant fungal groups and vegetation type are closely associated with the structure of those communities [33]. In this study, the three most abundant phyla accounted for up to 94.52% of the community, consistent with other studies of mountain soil fungal communities [34,35]. Although fungal community composition differed among vegetation types, Basidiomycota, Ascomycota, and Zygomycota remained predominant, consistent with findings by Xiao Ye et al. [36]. Ascomycota typically degrades hemicellulose and cellulose, whereas Basidiomycota produces lignocellulose-degrading enzymes that break down lignin [37]. Notably, Basidiomycota was more abundant in CB than in other vegetation types, a result that contrasts with Fan Yaqian’s findings [38]. The higher lignin content in CB soil likely favored Basidiomycota, which produce extracellular oxidases and hydrolases that degrade lignin. This process is associated with vegetation type. Yang Hu et al. [4] reported that Ascomycota and Basidiomycota dominated soil fungal communities across vegetation types on Helan Mountain’s slopes. Basidiomycota were markedly more abundant in forested areas, while Ascomycota dominated in GL and SL, consistent with our results. This distribution likely reflects the lower SMCs and better aeration of GL and SL soils, conditions that favor saprophytic Ascomycota that decompose organic matter, dead branches, and leaves. Variations in soil fungal community structures reflect distinct soil environments associated with different vegetation types.

This study examined the relationship between vegetation types and alpha diversity of soil fungi, revealing that GL exhibited significantly higher Shannon, Simpson, and Chao1 indices compared to DB. Notably, the DB community exhibited the lowest Chao1 index, a result that contrasts with findings by Feng Yongyu et al. in Liziping Nature Reserve [39]. This discrepancy may reflect that DB is a typical ecosystem in the middle to late stages of succession. As succession progresses, dominant plant species are observed to prevail, potentially suppressing subordinate plants and strengthening symbiotic associations between vegetation and fungi. This shift may reduce the available habitat for non-symbiotic fungi and thus lower fungal diversity. Overall, increased soil fungal richness enhances ecosystem stability and resilience to environmental change.

### 4.3. Co-Occurrence Network Topology and Biomarker Analysis

To further reveal the relationship between vegetation type and fungal community structure, network analysis was performed. Results showed that connectivity of fungal communities in GL soil is better than in DB, with a higher average clustering coefficient and average connectivity. Under the same number of nodes, GL has a greater number of connections, a higher average connection degree, and a higher average clustering coefficient than other vegetation types. This is consistent with previous research, indicating that fungal community structure in GL soil is more complex and potentially more robust than in forest vegetation types [40,41]. This difference in network structure further supports the association between vegetation succession and the organization mode of soil fungal communities.

In addition, biomarker analysis provides important evidence. Biomarkers are fungal groups significantly enriched in specific vegetation types and can reflect environmental differences [42]. It is worth noting that in CB, where DB and CF coexist, significant differences between the two plant types in root secretions and litter properties result in more diverse root secretions and nutrient substrates in the soil matrix, inhibiting growth of a dominant single fungal group. Therefore, a relatively small number of significantly different groups (i.e., biomarkers) were observed in the LEfSe analysis [43,44]. This discovery is corroborated by network analysis results, suggesting an association between vegetation type and the structure and interaction patterns of soil fungal communities.

### 4.4. Predicted Functional Potential of Fungal Communities

It is important to note that PICRUSt2 predicts metabolic potential rather than direct activity; thus, functional interpretations should be considered hypothesis-generating. Using the MetaCyc database, we assessed the functional diversity of soil fungal communities across vegetation types on the Jingpo Lake lava plateau. Results showed that primary metabolic pathways dominated these fungal communities. These pathways—including biosynthesis, precursor metabolite and energy production, and superpathways—constituted a synergistic system, consistent with previous studies [45]. The distribution of primary metabolic pathways and secondary metabolic functions was similar across vegetation types, indicating that the effect of vegetation on soil fungal functions was consistent. This consistency coincided with changes in soil physicochemical properties and in the soil microecological environment [46]. In lava plateau soils, which exhibit low SMC, strong phosphorus fixation, and nutrient deficiency, soil fungi may exhibit enriched genetic potential for biosynthetic pathways to preserve cellular homeostasis and adapt to low-phosphorus, high-oxidative stress conditions. Consequently, relative abundances of secondary metabolic functions, including de novo biosynthesis of adenosine ribonucleotides and fatty acid elongation, were predicted to be higher. This pattern aligns with adaptive strategies of fungi in tropical volcanic ash soils under low-phosphorus conditions [47]. The conditions in forest vegetation types, which exhibit rich biodiversity and complex ecosystem structures, are linked to soil fungal communities [48]. Spatial mismatches between biosynthetic functions and precursor metabolite or energy production were observed, and these mismatches were associated with root exudates from different vegetation types influencing fungal metabolism. In this study, relative abundances of methyl ketone biosynthesis and fatty acid oxidation I within degradation/utilization/assimilation peaked in CB. In contrast, in GL, significant differences in these two secondary metabolic functions reflected selective effects of different vegetation types on soil fungal ecological niches [46]. Different vegetation types are associated with functional differentiation among soil fungi, suggesting potential variations in their role in ecosystem nutrient cycling by supplying organic matter and creating a complex soil microecological environment.

### 4.5. Environmental Drivers of Fungal Community Variation

Multiple factors are associated with the soil fungal community structure, and their influences vary by region and vegetation type [49]. Previous studies have shown that the soil fungal community composition is influenced by pH [50], SOM [51], AP [6], and altitude [52]. In this study, SOM emerged as the dominant predictor of variation in fungal community structures and functional potential, closely associated with the abundances of Basidiomycota, Ascomycota, and Mucoromycota. Relative abundances of these phyla varied significantly across vegetation types, reflecting strong coupling among fungal communities, soil physicochemical properties, and vegetation characteristics [36]. SOM was strongly associated with fungal community composition. During the plant community succession, SOM, SMC, and K increased while pH and BD decreased across vegetation types; these changes were associated with enhanced microbial activity, improved soil physicochemical properties, and accelerated nutrient cycling, potentially strengthening ecosystem stability [53].

Soil fungal diversity is closely linked to ecosystem functional stability. In this study, it was found that as vegetation succession progressed, SOM [54], SMC [55], and N significantly [56] increased, while fungal diversity declined. This finding deviates from the traditional understanding that soil nutrient enrichment is simply positively correlated with fungal diversity, suggesting that the relationship may be nonlinear (e.g., hump-shaped pattern) and that the studied succession stages likely represent the declining limb where competitive exclusion dominates. In nutrient-rich forest environments, particular functional groups (for example, Basidiomycota) can attain competitive dominance and suppress other taxa, producing a simplified community structure. RDA confirmed that SOM is a key factor predicting fungal community diversity, with high explanatory power (R^2^ > 0.90), consistent with previous findings [57]. The elongation and saturation of fatty acids were strongly correlated with SOM. In environments enriched with these factors, the relative abundance of the fatty acid elongation pathway decreased, reflecting predicted fungal metabolic strategies tuned to differing nutrient regimes primarily associated with SOM, with moisture conditions as a secondary modulator. Conversely, microorganisms may possess the genetic potential for fatty acid elongation in nutrient-poor, water-limited settings to potentially better cope with those conditions. The pyruvate fermentation pathway for isobutanol production was correlated strongly with SOM and TN. This pattern suggests that, in soils rich in these components, microbes may have the genetic capacity to utilize this pathway, likely reflecting increased energy and carbon demands and greater substrate availability. Abundant organic matter and N supply the resources microorganisms need to carry out this fermentation, thereby suggesting a potential for isobutanol synthesis and potential production [58]. In this study, the analysis of correlations between metabolic pathway abundance and soil properties revealed complex interactions between predicted fungal metabolic functions and the soil environment.

## 5. Conclusions

Different vegetation types on the Jingpo Lake lava plateau are strongly associated with the soil’s physical and chemical properties and correspond to the fungal community structure. Ascomycota and Basidiomycota dominate the fungal assemblage in the study area, with *Sebacina*, *Cortinarius*, and *Mortierella* as the principal genera. Forest vegetation types exhibit relatively high soil nutrient and SMCs, which are significantly correlated with fungal community characteristics. The soil fungal community in GL showed greater network connectivity and modularity, while the fungal community structure across forest vegetation types was simplified, indicating that vegetation succession corresponds to changes in these structures alongside alterations in the intensity of interspecies interactions. In CB, the greater plant functional group diversity is associated with increased soil substrate heterogeneity and may limit dominance by a single group. The small number of biomarkers and the simplified network structure together support a mechanism in which vegetation types are associated with fungal community differentiation alongside variations in resource availability. The metabolic pathways of fungal communities across vegetation types are dominated by biosynthesis, precursor metabolite production, and energy generation. Vegetation is linked to soil fungal community structure and function alongside modifications in soil physicochemical properties, with interactions among vegetation, soil, and microorganisms mediating these effects. SMC is linked to nutrient contents and may facilitate the cycling and transformation of elements such as C, N, and P via organic matter mineralization, thereby facilitating plant growth. However, it may also cause nutrient loss. Therefore, forest vegetation types on the Jingpo Lake lava plateau represent multistage succession characterized by significantly lower soil fungal diversity, a simplified community composition, and more prominent biosynthetic functions. This illustrates how the belowground component of the advanced community responds to development of the aboveground vegetation. Through complementary ecological functions, these belowground processes support primary vegetation recovery on volcanic landforms, potentially optimize the efficiency of C, N, and P use, and contribute to enhancing soil fungal diversity. This study offers a novel scientific perspective on the processes associated with lava plateau ecosystem restoration and provides a theoretical reference for vegetation reconstruction and plant niche construction processes in volcanic landscapes. However, this study has limitations, including the use of PICRUSt2 for functional prediction (rather than metagenomic sequencing), single-time sampling, and the relatively limited replication (*n* = 3 per vegetation type), which may constrain the full characterization of within-type community variability; nevertheless, rigorous statistical methods (e.g., FDR correction, rarefaction to minimum depth) were applied to ensure result reliability, although causal relationships could not be established due to the observational design. Future studies should employ metagenomic or metatranscriptomic approaches, conduct long-term monitoring across multiple seasons, and utilize manipulative experiments to validate the causal mechanisms underlying vegetation–soil–fungi interactions in lava plateau ecosystems.

## Figures and Tables

**Table 1:** Dominant species of different vegetation types.

Vegetation Type	Sample Plot	Latitude–Longitude	Altitude (m)	Dominant Species
GL	GL1	128°56′79″ E–44°04′18″ N	355.0	*Artemisia mongolica*, *Vicia sepium*, *Campanula glomerata*, *Galium verum*
	GL2	128°56′03″ E–44°04′23″ N	356.0	
	GL3	128°56′64″ E–44°04′28″ N	361.2	
SL	SL1	128°56′76″ E–44°07′48″ N	357.3	*Lespedeza davurica*, *Lonicera maackii*, *Acer tataricum* subsp. *ginnala*
	SL2	128°56′80″ E–44°07′43″ N	358.4	
	SL3	128°56′81″ E–44°07′58″ N	360.2	
DB	DB1	128°58′10″ E–44°02′43″ N	416.2	*Populus davidiana*, *Quercus mongolica*, *Betula platyphylla*
	DB2	128°58′47″ E–44°02′46″ N	416.7	
	DB3	128°58′28″ E–44°02′49″ N	416.5	
CB	CB1	128°57′02″ E–44°02′23″ N	417.3	*Populus davidiana*, *Quercus mongolica*, *Betula platyphylla*, *Pinus koraiensis*
	CB2	128°57′71″ E–44°02′25″ N	417.8	
	CB3	128°57′83″ E–44°02′20″ N	417.0	
CF	CF1	128°58′68″ E–44°01′14″ N	417.3	*Pinus koraiensis*
	CF2	128°58′96″ E–44°01′15″ N	419.0	
	CF3	128°58′87″ E–44°01′19″ N	417.7	

Note: GL (grassland), SL (shrubland), DB (deciduous broad-leaved forest), CB (coniferous and broad-leaved mixed forest), and CF (coniferous forest).

**Table 2:** Soil physical and chemical properties for different vegetation types.

	BD (g·cm^−3^)	SMC (%)	pH	SOM (g·kg^−1^)	TN (g·kg^−1^)	TP (g·kg^−1^)	TK (g·kg^−1^)	AN (mg·kg^−1^)	AP (mg·kg^−1^)	AK (mg·kg^−1^)
GL	1.92 ± 0.06 ^b^	35.57 ± 0.71 ^c^	6.74 ± 0.21 ^a^	185.41 ± 2.22 ^d^	5.73 ± 0.16 ^c^	0.86 ± 0.11 ^b^	10.37 ± 0.15 ^b^	219.39 ± 4.69 ^e^	20.18 ± 1.44 ^ab^	126.36 ± 1.00 ^c^
SL	2.33 ± 0.24 ^a^	23.22 ± 2.50 ^d^	6.50 ± 0.22 ^ab^	188.17 ± 2.57 ^d^	5.88 ± 0.06 ^c^	0.96 ± 0.14 ^ab^	10.57 ± 0.06 ^b^	330.75 ± 4.83 ^d^	17.25 ± 1.74 ^b^	125.42 ± 3.25 ^c^
DB	1.88 ± 0.11 ^b^	52.12 ± 4.26 ^b^	6.34 ± 0.35 ^ab^	225.55 ± 2.57 ^c^	7.58 ± 0.21 ^b^	1.04 ± 0.05 ^ab^	10.63 ± 0.21 ^b^	474.72 ± 5.98 ^a^	23.39 ± 2.29 ^a^	175.38 ± 6.29 ^a^
CB	1.49 ± 0.18 ^c^	53.97 ± 3.45 ^b^	6.31 ± 0.19 ^ab^	246.20 ± 2.52 ^b^	7.66 ± 0.15 ^b^	1.13 ± 0.22 ^ab^	11.53 ± 0.31 ^a^	362.28 ± 5.28 ^c^	19.37 ± 1.89 ^b^	145.51 ± 7.08 ^b^
CF	1.01 ± 0.11 ^d^	66.75 ± 3.56 ^a^	6.23 ± 0.30 ^b^	253.01 ± 2.21 ^a^	8.49 ± 0.39 ^a^	1.25 ± 0.19 ^a^	11.77 ± 0.32 ^a^	440.91 ± 7.15 ^b^	20.28 ± 1.09 ^ab^	142.18 ± 1.95 ^b^

Note: Different letters in the same column represent significant differences at the 0.05 level. The same applies to Table 3. BD (bulk density), SMC (soil moisture content), SOM (soil organic matter), TN (total nitrogen), TK (total potassium), TP (total phosphorus), AN (available nitrogen), AP (available phosphorus), AK (available potassium). Data source: Zhang et al. [11].

**Table 4:** Topological structure of co-occurrence networks of soil fungal communities in different vegetation types.

Topological Feature	Fungus				
	GL	SL	DB	CB	CF
Node number	100	100	100	100	100
Edge number	1370	1037	963	1037	1065
Positive edge	725	613	712	641	689
Negative edge	645	424	251	396	376
Average degree	27.68	20.74	19.26	20.74	21.3
Average clustering coefficient	0.976	0.97	0.975	0.97	0.969
Mean distance	1.789	1.853	1.888	1.853	1.848
Network density	0.272	0.21	0.194	0.21	0.215
Modularity	0.624	0.659	0.713	0.653	0.668

## Data Availability

The original data presented in the study are openly available in the NCBI Sequence Read Archive (SRA) under the accession number PRJNA1327292.

## Abbreviations

The following abbreviations are used in this manuscript:

GL	Grassland
SL	Shrubland
DB	Deciduous broad-leaved forest
CB	Coniferous and broad-leaved mixed forest
CF	Coniferous forest
BD	Bulk density
SMC	Soil moisture content
SOM	Soil organic matter
TN	Total nitrogen
TK	Total potassium
TP	Total phosphorus
AN	Available nitrogen
AP	Available phosphorus
AK	Available potassium
ANOVA	One-Way Analysis Of Variance
RDA	Redundancy Analysis
PCR	Polymerase Chain Reaction
DNA	Deoxyribonucleic Acid
